# Cholera in New Orleans

**Published:** 1849

**Authors:** John C. P. Wederstrandt

**Affiliations:** New Orleans


### ARTICLE II.

### *Cholera in New Orleans.*

[The following is extracted from a letter addressed to Dr. A. Brigham, Superintendent of the State Lunatic Asylum, Utica, New York, by Dr. Wederstrandt, of the Charity Hospital, New Orleans. It was not intended for publication; but in the peculiar and threatening aspect which the Asiatic cholera has assumed in the Southwestern portion of our country, it is thought by Dr. Brigham, to whose kindness we are indebted for the letter, of sufficient importance for publication. It is the first and only account we have seen, from a reliable source, of the disease in New Orleans, and we therefore take the earliest opportunity of presenting it to our readers.---*Bost. Med. & Surg. Journ.*]

On the 12th of the present month, the cholera broke out in this hospital. The two first cases were a man and a woman, who were brought, in the last stage of the disease, from the ship Swanton, which had just arrived from Havre. This vessel left Havre with all the passengers and crew in good health, neither was the cholera in that port when she left; but some of the passengers were from a part of Germany where the cholera was raging. When at sea two weeks, the disease broke out on board, and 17 persons died in a few days, and were thrown overboard. At the time she reached here, but two were sick on board, and they were brought to this hospital. The very next day numerous cases appeared all over the city, but principally in the houses nearest to the shipping, or among persons employed on the wharves. Since the middle of this month [December] we have admitted between 40 and 50 persons with this disease every day; upwards of 50 cases have originated in the house among the convalescents of other diseases and the attendants; three of the washerwomen have taken the disease, and two have died. The disease here seems to consist of three stages in most cases: first a feeling of malaise and diarrhœa; next comes on the vomiting and purging of rice water discharges, and cramps; thirdly, the cold stage, with the clammy sweat and suppression of urine. The intelligence remains intact until very late. The disease has proved fatal here in so short a time as three hours. Oftener it is protracted to twelve and fifteen, and rarely beyond twenty-four hours. The violence of the pain in the stomach, and vomiting and purging, does not always afford a criterion for an unfavorable prognosis, for many patients recover rapidly in a few hours after being so attacked, declaring themselves nearly as well as ever. About half an hour after death, the body, which was as cold as ice just before, becomes as warm as in health; and the cramps or contractions of the muscles, which annoyed the patient so much during life, continue for at least half an hour, and in some cases nearly an hour, after death.

During the first days of the epidemic, nearly all the cases proved fatal; but within the last few days it seems to be rather on the decline, as our admissions and deaths have decreased, and we begin to number many cures, or rather recoveries. We treat the disease on general principles, and according to the indications of each individual case. In the early stage, we have had reason to be satisfied with the preparations of opium and counter-irritants. Some physicians use a large dose of opium and quinine in the beginning, when they get their cases early; they give from thirty to forty drops, and a drachm of tincture of opium, with half a drachm of quinine, for a single dose, and speak highly of their success. In a few cases I have thought that the practice did good, but I have not used it to any great extent. When brought to us, which they generally are, in the cold stage, we use stimulants, externally and internally, with nourishing broths, and several have re-acted under this treatment, and finally recovered. Males and females, young and old, are alike subjects for this disease; but far more men than women are attacked. We have seen many children die of it, some under five years, and a few old people at a very advanced age. Dr. Watson, in his very interesting and valuable Lectures on the Practice of Medicine, has given a most correct description of the disease as it now prevails among us, and I believe it to be identical with the Asiatic cholera, which he so ably describes.

I remain, very respectfully yours,

JOHN C. P. WEDERSTRANDT.

*New Orleans, Dec. 25th*, 1848.

